# Differences in Mobility and Dispersal Capacity Determine Body Size Clines in Two Common Alpine-Tundra Arthropods

**DOI:** 10.3390/insects11020074

**Published:** 2020-01-22

**Authors:** Niklas Beckers, Nils Hein, Alessa Anneser, Kim A. Vanselow, Jörg Löffler

**Affiliations:** 1 Department of Geography, University of Bonn, Meckenheimer Allee 166, D-53115 Bonn, Germany; nhein@uni-bonn.de (N.H.); s5alanne@uni-bonn.de (A.A.); joerg.loeffler@uni-bonn.de (J.L.); 2 School of Natural Sciences and Engineering, Ilia State University, 0162 Tbilisi, Georgia; 3 Department of Geography, University of Erlangen-Nuremberg, Wetterkreuz 15, D-91058 Erlangen, Germany; kim.vanselow@fau.de

**Keywords:** genus: *Amara*, species: *Amara alpina*, genus: *Pardosa*, species: *Pardosa palustris*, Bergmann’s rule, temperature–size rule, life-history, elevational gradients

## Abstract

The Arctic is projected to be severely impacted by changes in temperature and precipitation. Species react to these changes by shifts in ranges, phenology, and body size. In ectotherms, the patterns of body size clines and their underlying mechanisms are often hard to untangle. Mountains provide a space-for-time substitute to study these shifts along multiple spatial gradients. As such, mobility and dispersal capacity might conceal reactions with elevation. We test this influence on body size clines by comparing two common arthropods of the alpine tundra. We find that high mobility in the lycosid spider *Pardosa palustris* blurs elevational effects. Partially low mobility at least during development makes the carabid beetle *Amara alpina* more susceptible to elevational effects. Specific life-history mechanisms, such as brood care in lycosid spiders and holometabolic development in carabid beetles, are the possible cause.

## 1. Introduction

The Arctic is projected to exhibit severe changes in temperature and precipitation in the near- and long-term future [1]. Indeed, arctic ecosystems are already displaying marked responses to ongoing climatic and environmental changes [2,3,4]. In general, however, response mechanisms in arctic ecosystems remain poorly understood [5,6]. In-depth and long-term ecological monitoring remains limited to a few locations across the Arctic [7].

To overcome the scarcity of long-term climatic approaches, researchers have turned to mountains as a space-for-time substitution [8,9]. Along elevational gradients, environmental parameters vary over short distances, i.e., temperatures lapse at a rate of 5.5 K per 1000 m of altitude [10]. This makes elevational gradients suitable to infer on climatic changes across broader scales [10,11,12]. Local topography modulates these effects at the microscale [13] to generate a more complex picture [14,15]. It is therefore necessary to integrate these meso- and microscale gradients to identify the environmental drivers that shape species and ecosystem responses to climate change [12].

Of all taxonomic groups, invertebrates show the most striking declines in terms of their abundance and diversity worldwide [16,17]. While climatic drivers are not solely responsible for this decline [17]—especially in temperate zones, human land use is considered more important [16]—recent studies hint that climate change drastically affects invertebrates of the Arctic; key responses include altered phenology due to advances in onset of the growing season [3,18], distribution shifts [8,19], and changes in body size [3]. Gardner et al. [20] refer to changes in body size (of ectotherms) as the third universal response to anthropogenic climate change. However, they also note that results are not uniform despite a trend towards globally decreasing body size. Post et al. [6] stress the need to investigate possible causes for heterogenic ecological responses to climate change.

In any case, ectothermic species clearly adapt their physiology in response to changing environments [21,22,23]. Bergmann’s rule—in ectotherms more appropriately referred to as the temperature–size rule (TSR) [24]—predicts that body size decreases as environmental temperature increases. Higher temperatures result in increased metabolic rates, thus shorter development times, which in turn lead to a smaller body size [23]. Adapted for elevational gradients, this means that species become larger towards the poles and the top of the mountain [25]. However, season length decreases with elevation (or latitude). This may lead to an opposite pattern—decreasing body size with elevation (also known as the converse Bergmann’s rule) [21,23,25]. The underlying mechanisms are the generally limited time for foraging, growth, and development that lead to smaller animals [25]. These two patterns are nonexclusive and might thus obscure any observational trend in body size in the field. Species might even compensate for both effects, thus not displaying any trend. The absence of a trend is called countergradient variation [25]. As Chown and Klok [21] demonstrate, these three patterns occur even with closely related taxa and at close proximity. In this context, the problem of scale in ecology [12] becomes apparent. Bowden et al. [26] found that habitat heterogeneity at finer scales overrides effects at broader scales in species with low mobility. In turn, Hein et al. [27] and Horne et al. [28] recently proposed that high mobility in a species might obscure reactions to environmental factors at finer scales.

Our study aims to understand the effect of diverging species mobility and dispersal capacity in detecting and explaining body size variations along an elevational gradient. We observe patterns of body size for two common and highly abundant ectothermic arthropods in the alpine tundra. Whereas one species (Araneae, Lycosidae, *Pardosa palustris*) is highly mobile and has high dispersal capacities during all life stages, the other species in focus (Coleoptera, Carabidae, *Amara alpina*) shows lower rates of daily locomotory mobility [29,30] and dispersal capabilities (probably no flying, despite being winged [31,32]). Crucially, *A. alpina* undergoes phases of immobility during development (eggs, larval pupation) [30,33,34]. This contrasts with lycosid spiders—where adults provide brood care for both eggs and juveniles [35]. Therefore, we assume *A. alpina* to have a generally rather limited dispersal capacity and low rates of daily locomotory mobility compared to *P. palustris*, especially during early life stages. We test for shifts in body size of both species with elevation and the concomitant variation of environmental drivers. Our hypothesis is that higher mobility and dispersal capacity will obscure any effects of topography and elevation, suggesting that species with reduced mobility and dispersal capacity will be under higher pressure by environmental changes than species with higher mobility and different modes of dispersal.

## 2. Materials and Methods

Our study assesses data from the Long-Term Alpine Ecosystem Research project (LTAER-NO, e.g., [13]) in Southern Norway. The study area lies on the mountain massif of Mt. Blåhø (1618 m a.s.l.) in the central Norwegian Scandes at approximately 61° 54′ N, 9° 17′ E. According to Moen [36], the area is considered to be one of the most continental parts of Norway. The region has an annual precipitation rate of 300–400 mm in the valleys and up to 600 mm at the highest elevated sites [36]. Mean annual temperature is given by Löffler [37] at −0.7 °C at 1100 m a.s.l. and −2.7 °C at 1465 m a.s.l.

The alpine zonation can be described following Dahl [38]: Above the treeline, the low-alpine belt is covered mainly by dwarf shrub and heather vegetation (e.g., *Betula nana*, *Empetrum hermaphroditum* and *Vaccinium myrtillus*). Around 1350 m, a transition zone marks the gradual onset of the middle alpine belt [13]. The middle alpine belt is dominated by grassy vegetation (e.g., *Juncus trifidus*, *Carex bigelowii*, and *Luzula confusa*). With elevation, vegetation becomes increasingly patchy and is more often interrupted by areas of rocky debris and open soils. Generally, vegetation height is lower on ridges than on slopes. Ridges are also characterized by additional lichen cover, which is largely absent on slopes. Depressions are marked by species reflecting high soil moisture and wetness in general (e.g., *Sphagnum* ssp., *Eriophorum angustifolium*, *Rubus chamaemorus*).

The vegetational patterns in alpine-tundra habitats are a result of the pronounced seasonal dynamics of snow cover [2,26]. Snow cover generally increases with elevation. It is modulated in duration and thickness by topography [13,14]: The strong winter winds leave ridges within the alpine belt largely snow-free. South-facing slopes and depressions hold thicker covers of snow. Hence, they are particularly protected from frost during winter, but snow cover generally lasts longer on leeward (south-facing) slopes. During summer, they are also generally warmer and show higher maximum temperatures.

The sampling design covers an elevational gradient with 42 sampling sites from the treeline at approximately 1030 m a.s.l. to the peak of Mt. Blåhø. To account for possible effects of local topography, we sampled across the four characteristic topographical positions (ridges, depressions, south-facing slopes, and north-facing slopes). We installed three pitfall traps set 5–10 m apart from each other at each site. We sampled the whole summer season of 2010 in a fortnightly rhythm as soon as sites were free of snow. Sampling began in spring, first on a low-alpine ridge position on 25 April 2010. Snow cover lasted the longest at 1514 m a.s.l. (until 3 July 2010) on a north-facing slope. All traps were sampled within the same day. From the collected material, we derived measures of α-diversity. Elevation and position were recorded using differential GPS. At each site, we recorded the frequency of vascular plants in four plots of 1 m^2^, each with 25 subplots of 20 × 20 cm size (see Löffler and Pape [39] for a more detailed description).

The design of the pitfall traps generally follows Ellenberg [40]. They consist of a glass container of 120 mm height and an opening diameter of 55 mm. A semitransparent roof is placed approximately 5 cm above the trap. Saturated salt solution was used for preservation in the traps and Agepon^®^ was added to reduce surface tension. All samples were later preserved in 70% ethanol. The material is stored at the Department of Geography at the University of Bonn, Germany.

Pitfall trapping has proven to be reliable, especially in alpine habitats [41,42,43,44]. Practical aspects, i.e., the possibility to create large and systematic datasets using a passive method, make this a widely used method [34]. However, the method has received pronounced criticism [45,46]. The interpretation of catch rates demands careful interpretation: Catch rates of pitfall traps are measures of species’ activity and local abundances (the concept of "activity–abundance") [30,47]. Therefore, the method is biased to a) species with a higher general mobility and size [45,48], and b) more active individuals within the same species (e.g., males) [46]. Moreover, low permeability of some habitats limits species mobility and therefore catch rates [49]. Despite these downfalls, the method can produce reliable estimates of species richness at a site [50] (especially for wandering spiders) and overall populations of ground-dwelling arthropods such as carabid beetles, harvestmen, and spiders [41,49,51]. Our sampling should result in a comparable data set, because the previous mentioned constraints are systematically present at all sites. The present study does not focus on the aspects of catch rates per se but rather intraspecific body size patterns. To balance the sampling efforts between sites as a result of season length, we standardized catch rates to periods of 100 trapnights [42]. We used the catches of the respective species as explanatory variables in our analysis as a measure of intraspecific competition (i.e., does local abundance affect body size?).

We assessed body sizes by measuring specific body parts for the two species. Both species coexist without a significant effect of predation of one another [30]. Both species are considered generalists in alpine habitats.

The wolf spider *Pardosa palustris* (Linnaeus 1758) is a widely distributed species across the northern hemisphere [52]. It is commonly found in open, moist to dry habitats up to 2500 m a.s.l. [43,52]. In the study area, it occurs in high numbers from the treeline up to 1534 m a.s.l. [43]. Lycosid spiders, in contrast to most carabid beetles, display brood care in the sense that they carry their egg sack fixed to their spinnerets until hatching. Hatched juveniles are then carried on the abdomen of the female as she wanders around the habitat [35]. *Pardosa* sp. are considered generalist wandering predators [53,54], feeding opportunistically on other invertebrates. It can be assumed that in the harsh environments of the arctic tundra, prey is chosen mainly based on their abundance, with little sign of niche segregation between dominant spider species [55]. The locomotory mobility rates of adult *Pardosa* species can lie within the range of several hundreds of meters per day [53,56,57]. Ballooning is a common but passive dispersal trait of spiders. In some cases, ballooning spiders can cover large distances and reach remote areas, e.g., islands. This trait, however, results in random displacement of the passively transported individuals [58]. *Pardosa palustris* commonly has a univoltine life-cycle, with the first spiderlings hatching from the egg sacs in June and commonly overwintering at an older instar level or subadult stage in temperate regions [59]. Generally, *Pardosa* species do not overwinter in the adult stage [60]. As a response to the short summer seasons at higher elevations in arctic-alpine environments, *P. palustris* obviously has a prolonged life-cycle of 2–3 years [61]. This results in individuals overwintering in a subadult stage, which can quickly reach adulthood in spring, when conditions are favorable. The advantage of the prolonged life-cycle at high elevations is thus to grow larger and have higher reproduction values, always under the disadvantage of a concomitant increased mortality rate [62]. So far, our previous studies on *P. palustris* in the research area studies could not unravel whether *P. palustris* shows a prolonged life-cycle at higher elevations or not [27,63,64]. In females, however, the production of egg sacs might be delayed at higher elevations and latitudes due to the timing of snow melt [63,64]. Specimens of *P. palustris* are active right after snow melt, which is highly variable depending on the sampling site, but commonly starting at the end of May and the beginning of June in our research area. Adult males can then be found until the end of July beginning of August, while adult females are active during the whole summer season [43].

The ground beetle *Amara alpina* Paykull 1790 is a typical species of alpine habitats [31,65] and occurs along the entire elevational gradient [44]. Ottesen [65] found that *A. alpina* does not seem to favor drier soils, as other species of the genus seem to do [30,33]. Imagos of *A. alpina* are omnivores: Their diet consists (likely in equal parts in terms of biomass [66]) of animal prey and plant biomass, including bryophyte mosses and seeds [66,67]. *A. alpina* is a common inhabitant of open habitats within the alpine-tundra environment [41,65,67]. In the alpine belt it is often a dominant species in studies based on pitfall catches [41]. *A. alpina* can be either macropterous (i.e., wings are fully developed) or brachypterous (reduced hind-wings) [33]. In our study, we observed no brachypterous animals. However, we never observed flying animals in the field. It can be assumed that flying is—at best—a sporadic mode of dispersal [30,31,34]. Running on the ground is the most common mode of mobility in any case. Distances covered by animals are likely to be less than 20 m per 24 h [29,30].

In principle, *Amara alpina* probably follows a univoltine life-cycle [31]. There is a clear peak in activity of the adults in midsummer [31,41,44]. At this time, the animals copulate, lay their eggs [68], and many of the adults perish shortly after [31]. Larvae probably hatch in the late summer/early fall [68]. Adults have been shown to hibernate during winter [68]. However, larval overwintering (in the last instar) and consequent (quick) maturation in the following spring is also possible [31,68,69]. The life-cycle may thus be very flexible [70]. Hibernation of both developmental stages means that these species are well-equipped to act as colonizers and pioneers in the alpine [68,71,72]. In our study area adults of *A. alpina* emerge right after snow melt, with males appearing earlier than females. Following Andersen [69] and Hågvar et al. [68], we assume that *A. alpina* commonly takes up to two years to complete its life-cycle in our study area. This prolonged life-cycle is obviously an adaptation to life in the alpine-tundra environment with generally shorter seasons [69]. We currently have no insight whether the life-cycle might change along the elevational gradient. It is likely that life-cycles may be shorter at favorable conditions or during warm periods [69]. This would suggest a higher ratio of adult hibernation at favorable sites/times in contrast to harsher conditions. So far, our insights into adult phenology give no indication in this regard—for example, a delayed peak activity at higher elevations. Instead we find stable peaks of activity in midsummer at all elevational levels.

To find proxies for body size, we identified species-specific variables that proved to be reliable measures in morphometric studies. Regarding the measurements of body size, preservation in alcohol sometimes leads to distortions of soft tissues and rigidness [33]. We followed Hågvar et al. [68] and chose pronotum size as a proxy for overall body size in *A. alpina*. In this species, the base of the pronotum is straight and marked with a “tooth” at the lateral ends. This part of the body is therefore appropriate to generate a consistent measure across many specimens [73]. Preliminary investigations showed pronotum width at the base correlated strongly with pronotum length across the middle (R = 0.92, *n* = 50) and elytra length (R = 0.91, *n* = 50). In lycosid spiders, the unsclerotized abdomen is rather soft and prone to damage [27]. Damage is often unavoidable in species determination of spiders. Especially female spiders are usually determined to species via dissection of their genitalia, which leads to damage to the abdomen. Measuring the width of the sclerotized prosoma is a viable proxy for overall body size [27,74]. For these reasons, we will use body size synonymously with both proxies when we describe our own data set.

We measured prosoma/pronotum widths using high resolution digital images of the specimens. Digital photographs were made using a stereomicroscope at 56–100× magnification. All specimens were aligned horizontally on a small patch of sand within a Petri dish. Body length was then measured using ImageJ software [75]. All measurements were adjusted to scale.

In our statistical analysis, we followed the data exploration protocol proposed by Zuur et al. [76]. We excluded collinear predictor variables: Variance inflation factors (VIF) were calculated repeatedly and the variable with the highest score was removed until all remaining variables scored <3 [76]. Our experiment lacks independent spatial replication. Therefore, we must account for spatial autocorrelation in our analysis. Furthermore, arthropods can display sexual size dimorphism. We chose a mixed model approach using site and sex as random effects to account for these biases. Linear mixed effect models [77] were fitted to explain the influence of spatial parameters (elevation, topography), α-diversity of vascular plants, and intraspecific competition at each sample (per individual trap) and per site (three traps per site as described above; Table 1). Akaike’s information criterion (AIC) [78] was used to optimize our model setup as follows: First, we set up a linear model using all non-collinear predictors and derived the AIC score. This served as a baseline comparison to base our model selection. We then calculated a linear mixed effect model using the function lmer in the R package lme4 [77]. The use of linear models demands (at least approximated) normality of the data [76]. We evaluated our models by calculating goodness-of-fit [79] as marginal R^2^ (R^2^m—the goodness-of-fit associated with only the fixed effects of the model) and conditional R^2^ (R^2^c—the goodness-of-fit of fixed and random effects). We optimized our model by a stepwise reduction of variables based on the AIC to achieve an optimal fit. In total, we calculated six models to explain body size patterns (Model 1: all specimens of *A. alpina*, Model 1M: male specimens of *A. alpina*, Model 1F: female specimens of *A. alpina*, and Models 2, 2M, and 2F, respectively, for *P. palustris*).

We identified the importance of the explanatory variables by using a 10×-repeated, 10-fold cross-validation of the model using the R package sperrorest [80,81]. The method includes permutating each predictor 100 times while keeping all other components constant. In effect, this increases the model prediction error (root mean squared error, RMSE). The increase in RMSE is higher when an important variable is arbitrarily modified. Variables which do not or only slightly increase the RMSE can be considered unimportant [44]. We used the R environment [82] for all statistical analyses.

## 3. Results

### 3.1. Body Sizes Per Species

**Figure 1:**
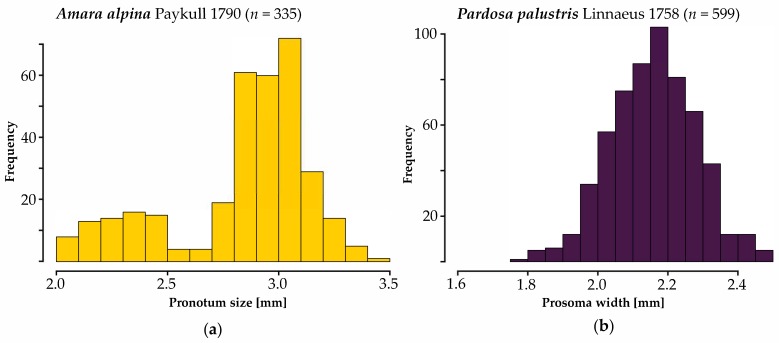
Frequency histograms of pronotum width (*Amara alpina*, (**a**); n = 335) and prosoma width (*Pardosa palustris*, (**b**); n = 599) in this study. The color scheme (*A. alpine*—yellow, *P. palustris*—dark blue) is used in subsequent figures to enhance interpretation.

We measured pronotum width in a total of 335 specimens of *Amara alpina*. The mean pronotum width in our study is 2.84 mm, with a standard deviation (hereafter SD) of 0.31 mm. Females are slightly larger than males: Females have a mean pronotum width of 2.93 mm (SD = 0.3, *n* = 142), while male specimens have a mean pronotum width of 2.77 mm (SD = 0.31, *n* = 193). The largest specimen is a female (3.43 mm) caught on a ridge position at 1035 m a.s.l. in early September. The smallest specimen is a male (2.03 mm) caught on a north-facing slope position at 1390 m a.s.l. in July. Figure 1a shows that pronotum width deviates from a normal distribution. A portion of animals is significantly smaller than the mean, while deviation from the mean is less pronounced when animals become larger.

We measured prosoma in a total of 599 specimens of *Pardosa palustris*. The mean prosoma width is 2.16 mm (SD = 0.12; Figure 1b). The sexual size dimorphism is negligible: Females have a mean prosoma width of 2.16 (SD = 0.14, *n* = 282). Male specimens have a mean prosoma width of 2.15 mm (SD = 0.1, *n* = 317). The difference between the sexes is less than the standard deviation in either case. The biggest specimen is a female (2.5 mm) caught on a north-facing slope at 1305 m a.s.l. in early July. The smallest specimen is also a female (1.76 mm) caught at 1074 m a.s.l. in early June. Figure 1b shows that the data follow a normal distribution around the mean.

### 3.2. Environmental Drivers of Body Size

We calculated models using sampling site and sex as random effects when focusing on all specimens of the respective species (sex only in models of all specimens of a species, as this would obviously not lead to sensible results when modeling, e.g., only males). Thus, we were able to eliminate effects of spatial autocorrelation.

In general, our models performed reasonably well given some limitations. RMSE values are well below standard deviation of body size in all models of *Amara alpina* and about the same order as SD in *Pardosa palustris*. However, linear mixed effects models are clearly a compromise in models 1, 1M, and 1F (*A. alpina*). The reason for this lies in the skewness of the distribution of pronotum width (Figure 1a), i.e., the distribution does not conform to normality. Nevertheless, deviation from the expected mean of the model is within acceptable margins (Appendix A) and deviation is symmetric on both ends. To ensure a succinct and comparable result between both species, especially regarding cross-validation of the models, we decided against using, e.g., generalized additive modelling or data transformations. The final models are listed in Table 2 for *A. alpina* and Table 3 for *P. palustris*.

**Figure 2:**
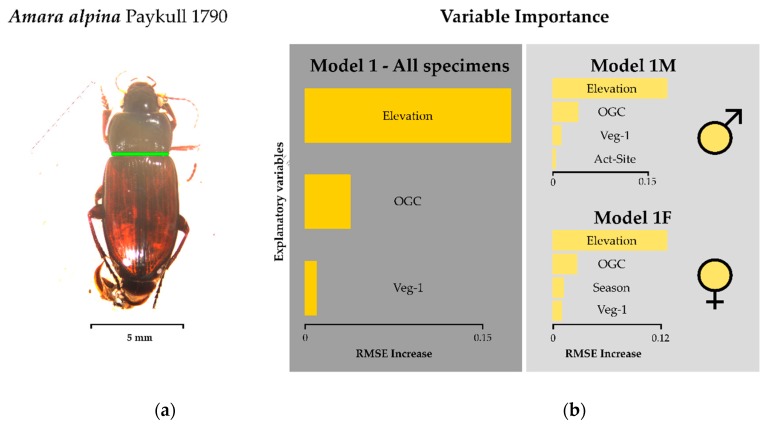
Model results for pronotum width: (**a**) green bar on photograph indicates location of measurement, (**b**) variable importance determined by RMSE increase of model permutations) of the ground beetle *Amara alpina*. See Table 2 for model formulation and Table 1 for a detailed description of model parameters. Variables used here are Elevation, OGC = open ground cover, Veg-1 = number of plant species, Act-Site = activity–abundance of *A. alpina* per site. Results are given for all specimens (dark grey field) and based on sex (light grey field).

Elevation is the most important predictor of body size in *A. alpina* in this study. This is true for either sex as well as all specimens combined (Figure 2). Body size decreases with elevation. This seems to be a result of a number of extremely small animals above approximately 1390 m a.s.l. (Figure 1a). The percentage of open soil is a second important variable, which has a negative effect on body size. The effect of other predictors is negligible.

**Figure 3:**
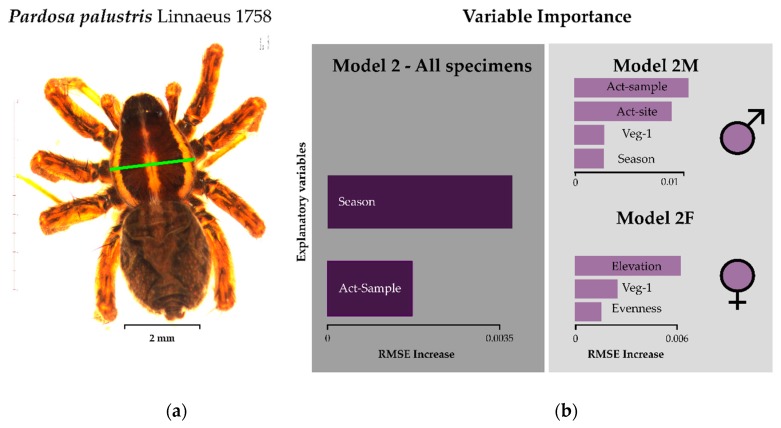
Model results for prosoma width: (**a**) green bar on photograph indicates location of measurement, (**b**) variable importance determined by RMSE increase of model permutations) of the wolf spider *Pardosa palustris*. See Table 3 for model formulation and Table 1 for a detailed description of model parameters. Variables used here are Season = day of the year, Veg-1 = number of plant species, Evenness = evenness of plant species per site, Act-Site = activity–abundance of *P. palustris* per site, Act-sample = activity–abundance of *P. palustris* per individual sample. Results are given for all specimens (dark grey field) and based on sex (light grey field).

**Figure 4:**
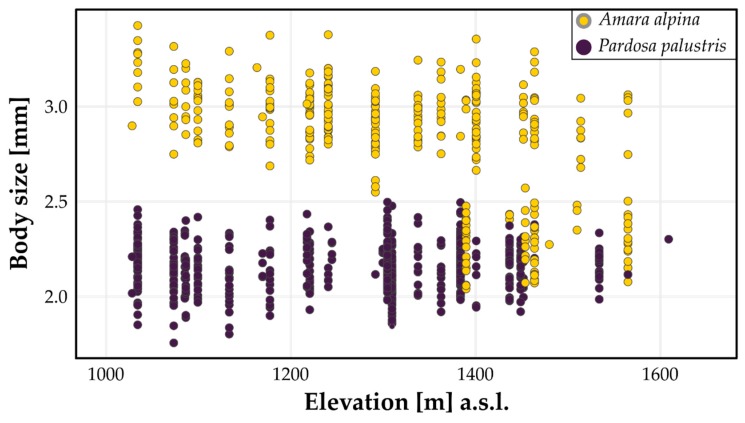
Body size patterns of *Amara alpina* (yellow marks) and *Pardosa palustris* (purple marks) along the elevational gradient.

We did not find clear drivers of body size for *P. palustris*. RMSE increase in the cross-validation was negligible, while the overall RMSE of the model was as high or slightly higher than the SD of body size. This suggests overall poor model fit and inconsistent results between the models. We did not find a consistent signal between models 2, 2M, and 2F (Table 3, Figure 3). Figure 4b illustrates that elevation does not have any significant effect on body size in any model of *P. palustris*.

## 4. Discussion

Our results imply that species’ mobility and dispersal capacity determine whether body size clines can be observed along elevational gradients. While we did detect a clear elevational signal for the ground beetle *Amara alpina*, we could not confirm this for the wolf spider *Pardosa palustris*. This meets the assumption of our hypothesis. We thereby confirm the proposed effect of mobility at the observational level noted by Hein et al. [27] and Bowden et al. [26].

There are several possible explanations for the exact mechanism behind the decrease in body size of *A. alpina*. The species’ life-history is certainly the most striking difference to the spider *P. palustris*. We want to propose three possible explanations for this finding (which are nonexclusive): The first direction of the argument turns to the mobility during the early life stages of *A. alpina*: Egg and larval stages of Carabidae are particularly vulnerable in extreme climatic conditions [30,65]. Saska and Honek [83] show that both stages strongly depend on thermal constants to complete development. Poor diet increases the days above a lower thermal threshold, which is needed for optimal development. This implies that instars from *Amara* species suffering from food scarcity— as they might do at high elevations and shorter season lengths—are more vulnerable to lower temperatures. They have to adapt by entering quiescence—a short period of externally forced inactivity [30]. This might explain the nonlinear pattern we found for *A. alpina* body sizes in our study: In the middle-alpine belt, mostly at north-facing slopes, we detected a bundle of extremely small specimens. This coincides with longer overall snow cover, i.e., shorter seasons and lower overall temperatures during summer at these sites [44]. It may be that the combination of low temperatures and food scarcity (resulting from reduced foraging time as a function of quiescence) approaches a tolerance threshold. While development can still be completed, it is hindered by the adverse conditions. Above 1565 m a.s.l., we find no more specimens of *A. alpina,* indicating that *A. alpina* cannot cope with the harsh environment at high elevation. We detect no adult specimens since development cannot be completed. Nevertheless, this might be the case in favorable years. In this regard, it might be interesting to compare our findings with results from glacial succession studies, where *A. alpina* is a characteristic pioneer on nutrient-poor young moraines [66,67,84] and where feeding habits can switch as an adaptation to food availability [85]. The sudden drop in body size might also indicate a switch from predominantly adult/teneral to larval hibernation as third instars, i.e. a switch from a univoltine to a semivoltine (biennial) life-cycle [86]. However, the knowledge on the larval biology of *A. alpina is limited*—a product of a probably cryptic life-style of the larvae [68,70]. Obviously, fundamental gaps of knowledge remain concerning life-cycle shifts and their environmental drivers, hibernation and larval biology, even in generally well-studied taxa such as Carabidae.

A second possible explanation follows the work of Schmidt (as summarized in [30]): Carabidae probably do not sense unfavorable temperatures directly but primarily seek to regulate their transpiration. They tend to migrate to sites where transpiration rates are lower. In many species the transpiration rate increases with decreasing humidity and higher temperatures. Thus, cold-adapted species, such as *A. alpina* [30,44,87], may seek shelter from wind and high insolation at the cooler, north-facing slopes. However, this means that body size decreases as a function of the abovementioned effects of season length. Alternatively, this strongly reduced body size is a direct adaptation to increased transpiration risks due to wind exposure (lower humidity) at higher elevations.

Thirdly, species locomotory mobility (daily activity) and dispersal capacity of the adults differ immensely between carabid beetles and lycosid spiders [27,29,30,57]. This means that adults of *A. alpina* are less capable to react to unsuitable conditions via evasion. Fertile adults, especially gravid females [30,34], are improbable to fly. Thus, they are likely forced to reproduce and/or lay their eggs in unfavorable sites. Because body size also determines fecundity and dispersal capacity [30], subsequent generations gradually become smaller if there is little exchange between populations of different elevations. Hence, the nonlinear pattern might represent two different populations within close distances. Simon et al. [88] recently found this to be the case for high arctic aphids: Poor dispersal capacity—an adaptation to the harsh environment on Svalbard—leads to genetically distinctive subpopulations over a few hundred meters. Similar results come from Arthofer et al. [89] who suggest the existence of three different cryptic species for the harvestman *Mitopus morio* in a mountain habitat. However, the data basis on the exact locomotory mobility, and even more so on flying, is especially scarce on *A. alpina*. Capture–recapture experiments would provide further insights into this phenomenon.

Our results for body size indicate no linear trend with elevation in *P. palustris*, even though body size adaptations in response to elevation are a well-described phenomenon in some *Pardosa* species [74,90]. Our result, no clear linear trend in body size of *P. palustris* along the elevational gradient, is in line with various previous findings on the body size variation of *P. palustris* in the research area [27,63,64]. Several reasons are considered to be responsible for the lack of an elevational trend. Firstly, the uneven distribution of competition along the elevational gradient might be the reason for the nonlinear pattern in body size. Previous studies showed that the smallest individuals of *P. palustris* are commonly found at the transition zone between the low- and middle-alpine belts [63,64]. The transition zone represents the middle of our elevational gradient. Here, high spider species number [43] and thus high competition between species over resources might result in the lowest body sizes in *P. palustris* [64]. Secondly, even though our study design covers an elevational gradient from the treeline up to pronounced middle-alpine conditions, the extension of only 500 m might be too short to find a change in body size. In this context, Wundram et al. [14] showed that increasing elevation does not necessarily lead to unfavorable conditions for ground-dwelling arthropods. The lack of higher and denser vegetation at higher elevated sites can lead to higher solar irradiance at specific sites, which results in higher temperature values at high elevated sites compared to lower ones. This implies, however, that a species shows relatively high mobility rates and behavior to avoid unfavorable sites. Hein et al. [27] found the high mobility of *P. palustris* during all life stages to be the most likely factor resulting in the lack of a clear pattern towards higher elevated sites and unfavorable conditions in the research area. Additionally, the high dispersal capability by means of ballooning in *P. palustris* conceals the detection of a linear elevational trend.

Our results have implications for conservation biology. It is generally assumed that species at higher latitudes are more resilient towards a global temperature increase, because they have a broader thermal tolerance spectrum [91]. Our results indicate that this is not always the case. Alpine-tundra ecosystems contain species that are resilient to unfavorable conditions: *P. palustris* can react by spatial evasion, either as adults or carrying their offspring to more favorable habitats. Hence, it is likely that these species will even profit from the globally changing environmental conditions in terms of their pan-Arctic distribution. Species like *A. alpina*, which are less mobile especially during development, are more susceptible to unfavorable conditions. In this context, Turin and Den Boer [92] found that species with a poor dispersal capacity have seen decreasing trends when analyzing museum samples. In contrast, strong dispersers even increased in abundance. However, as Hallmann et al. [16] note, in central Europe this is more likely a result of land use and not the change in climatic conditions. Nevertheless, our results highlight that the aspects of species biology need to be included in modelling species responses to climate change. This concerns above all the effect of life-history on mobility and dispersal and their implications for extinction risks [93]. Moreover, we demonstrate that crucial gaps of knowledge still exist on a species level. This is even more striking considering both our model organisms belong to taxonomic groups which are rather well studied (in comparison to, e.g., their most important shared prey—Collembola [30,34,66]).

## 5. Conclusions

We show that mobility and dispersal capacity govern the appearance of body size clines along elevational gradients. In our study, this is mainly a result of the biology of early life stages in two alpine-tundra species. We demonstrate that species with stationary larval and egg stages are more susceptible to unfavorable climatic conditions than species which display brood care. Our study also demonstrates the need for further studies on early phases of life-history of arthropods and their biology in general. Considering our results, these data are urgently needed to improve the quality of predictions on species responses to climate change.

## Figures and Tables

**Table 1:** Model parameters used in this study to explain the patterns of body sizes in the ground beetle *Amara alpina* and the wolf spider *Pardosa palustris*. Final models were selected by stepwise deletion of fixed effects based on Akaike’s information criterion. Collinear variables were not included in this study.

Variable	Type	Response/Explanatory	Description
Size [mm] ^1^	-	Response	Pronotum width in *Amara alpina*, prosoma width in *Pardosa palustris*
Elevation [m] a.s.l.	Spatial	Fixed effect	~1030–1618 m a.s.l.
Topography	Spatial	Fixed effect	Four positions (ridge, depression, south-facing slopes, north-facing slopes)
Season	Temporal	Fixed effect	Day of snowmelt (at each site)
Veg-1	Biotic	Fixed effect	Abs. number of plant species per site
Evenness	Biotic	Fixed effect	Evenness of plant species frequency per site
Open Ground Cover (OGC)	Biotic	Fixed effect	Percent of open ground per site
Act-Site	Biotic	Fixed effect	Activity–abundance ^1^ of the respective species at each site through the season
Act-Sample	Biotic	Fixed effect	Activity–abundance ^1^ of the respective species for each sample within the season
1 | Site	-	Random effect	UTM coordinates of the site
1 | Sex	-	Random effect	Sex of the specimen (only used in models using both sexes, i.e., all specimens of a species)

^1^ Corrected to 100 trapnights for sampling effort.

**Table 2:** Model selection to explain the patterns of body size in the ground beetle *Amara alpina*. Final models were selected by stepwise deletion of fixed effects based on Akaike’s information criterion (AIC). Collinear variables were not included in this study. RMSE = root mean squared error, R^2^m = marginal R^2^, R^2^c = conditional R^2^; see Table 1 for a detailed explanation of the variables.

Model	Type	Response	Predictor Variable		AIC	RMSE	R^2^m	R^2^c
			Fixed	Random				
Model 1 ^1^	Baseline	All	All variables	-	−935.0	0.24	-	-
	Final	All	Elevation + Veg-1 + OGC	1 | Site 1 | Sex	−74.5	0.23	0.3	0.64
								
Model 1F^1^	Baseline	Females	All variables	-	−395.1	0.24	-	-
	Final	Females	Elevation + Veg-1 + OGC + Season	1 | Site	−1.8	0.3	0.36	0.53
								
Model 1M^1^	Baseline	Males	All variables	-	−550.1	0.23	-	-
	Final	Males	Elevation + Veg-1 + OGC + Act-sample	1 | Site	−47.7	0.28	0.35	0.65

^1^ Final model is not the better fit but used because of random effects to correct for spatial autocorrelation and sexual size dimorphism.

**Table 3:** Model selection to explain the patterns of body size in the wolf spider *Pardosa palustris*. Final models were selected by stepwise deletion of fixed effects based on Akaike’s information criterion (AIC). Collinear variables were not included in this study. RMSE = root mean squared error, R^2^m = marginal R^2^, R^2^c = conditional R^2^; see Table 1 for a detailed explanation of the variables.

Model	Type	Response	Predictor Variable		AIC	RMSE	R^2^m	R^2^c
			Fixed	Random				
Model 2 ^1^	Baseline	All	All variables	-	−2530.8	0.12	-	-
	Final	All	Season + Act-sample	1 | Site 1 | Sex	−838.5	0.13	0.02	0.1
								
Model 2F^1^	Baseline	Females	All variables	-	−1112.8	0.14	-	-
	Final	Females	Veg-1 + Evenness + Season	1 | Site	−313.93	0.14	0.03	0.07
								
Model 2M^1^	Baseline	Males	All variables	-	−1439.2	0.1	-	-
	Final	Males	Veg-1 + Season + Act-sample + Act-site	1 | Site	−548.9	0.15	0.06	0.16

^1^ Final model is not the better fit but used because of random effects to correct for spatial autocorrelation and sexual size dimorphism.

**Figure A1:**
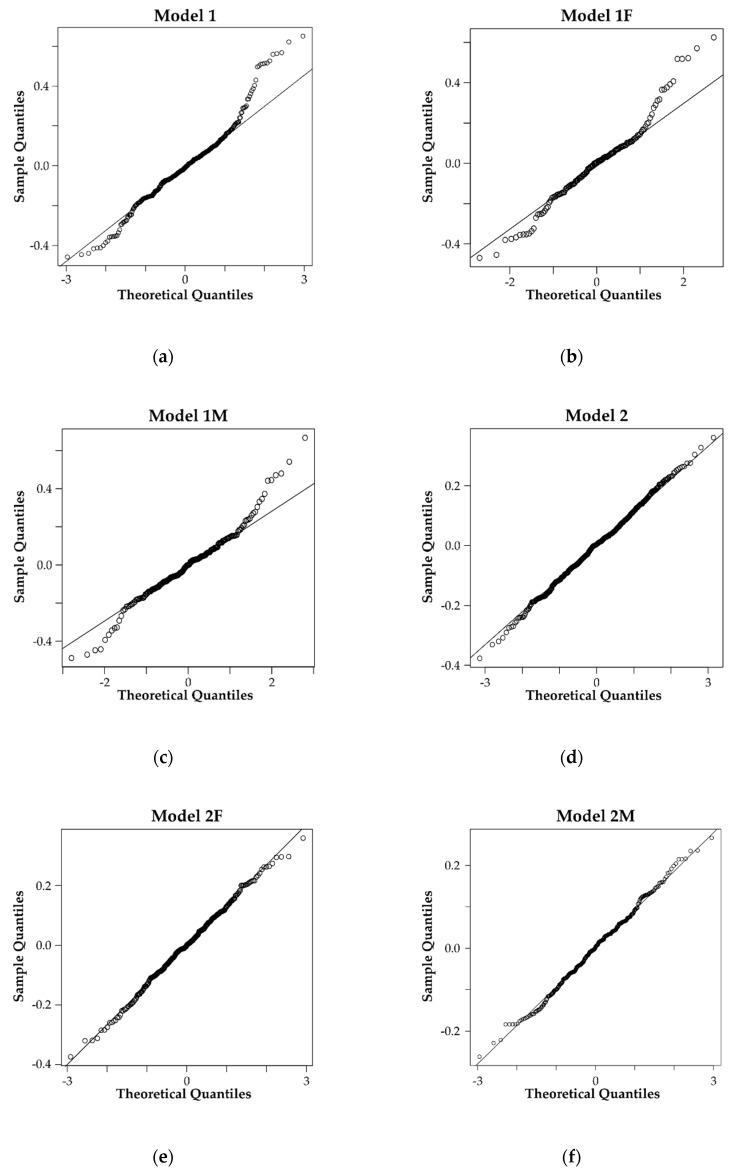
Q-Q plots of residuals of the linear mixed effects models used in this study. Models were calculated for all specimens of *Amara alpina* (**a**—Model 1), females of *A. alpina* (**b**—Model 1F), males of *A. alpina* (**c**—Model 1M), and all specimens (**d**—Model 2), females (**e**—Model 2F) and males (**f**—Model 2M) of *Pardosa palustris*, respectively. The expected mean of residuals is plotted as a line.
